# Which Patients With Dysfunctional Voiding Respond Well to Sacral Neuromodulation? ICI‐RS 2025

**DOI:** 10.1002/nau.70185

**Published:** 2025-11-26

**Authors:** Jalesh N. Panicker, Chris Harding, Hashim Hashim, Claire Hentzen, Nikita Bhatt, Arjun Nambiar, Brigitte Schurch, Mathijs de Rijk, Stefania Musco

**Affiliations:** ^1^ Department of Uro‑Neurology, The National Hospital for Neurology and Neurosurgery University College London NHS Foundation Trust London UK; ^2^ Department of Translational Neuroscience and Stroke, UCL Queen Square Institute of Neurology, Faculty of Brain Sciences University College London London UK; ^3^ Department of Urology Freeman Hospital, Newcastle upon Tyne UK; ^4^ Translational and Clinical Research Institute Newcastle University UK; ^5^ Bristol Urological Institute Southmead Hospital Bristol UK; ^6^ GREEN Group of Clinical REsEarch in Neurourology, AP‐HP, Hôpital Pitié Salpétrière Sorbonne University, GRC 01 Paris France; ^7^ Neurourology Unit, Department of neurosciences Université Hospital Chuv Lausanne Switzerland; ^8^ Department of Urology, Mental Health and Neuroscience Research Institute, Faculty of Health, Medicine and Life Sciences Maastricht University Maastricht the Netherlands; ^9^ Department of Urology Maastricht University Medical Centre + Maastricht the Netherlands; ^10^ Neuro‐Urology, Department of Skeletal Muscle and Sense Organs Azienda Ospedaliero Universitaria Careggi Florence Italy

**Keywords:** dysfunctional voiding, Fowler's Syndrome, pressure flow study, sacral nerve stimulation, sacral neuromodulation, urinary retention, voiding dysfunction

## Abstract

**Aims:**

Dysfunctional voiding (DV) is characterised by fluctuating or intermittent urinary flow during voiding in neurologically normal individuals. Given the different definitions used and heterogeneous pathophysiologies, outcomes following sacral neuromodulation/sacral nerve stimulation (SNM/SNS) are variably reported. The aim was to identify the areas of research required to be able to accurately predict response to SNM/SNS in adults with DV.

**Methods:**

The relevant literature was reviewed by a multidisciplinary panel and the findings were discussed at the ICI‐RS meeting held in 2025 in the UK. The outcomes of this discussion are presented.

**Results:**

DV has unique diagnostic features, typically requiring pressure‐flow studies and uroflowmetry to establish the diagnosis. Further investigations such as electromyography and urethral pressure profilometry help to better understand the pathophysiology. Phenotyping the lower urinary tract dysfunction helps to identify patterns of abnormalities, and it is likely that certain groups show a better response to SNM/SNS than others, such as in Fowler's syndrome. Older age and change in body weight following implantation are associated with a worse outcome, though not specifically in DV. Studies evaluating the impact of neuropsychiatric co‐morbidities on SNM/SNS outcomes show mixed results. Central dysregulation of micturition networks possibly contribute to DV and could represent an additional therapeutic target of SNM.

**Conclusion:**

Precise phenotyping of individuals with DV integrating clinical, urodynamic, neurophysiological, and neuropsychiatric factors is essential to predict which adults respond best to SNM/SNS. Future research should focus on establishing criteria for patient selection and designing comprehensive prospective interventional studies to assess efficacy and complications.

## 1 Introduction

Dysfunctional voiding (DV) is characterised by abnormal patterns of voiding in the absence of overt neurological disease or anatomical obstruction [1]. The term has been used inconsistently across studies and is often erroneously interchanged with voiding dysfunction. The lack of clarity in the definition has been a major obstacle to standardising investigations and establishing a diagnosis. Interventions range from behavioural and physiotherapy strategies to more invasive approaches such as sacral neuromodulation (SNM), also referred to as sacral nerve stimulation (SNS). The variability in reported outcomes reflects the inconsistencies in definitions, patient phenotyping and assessment methodology [2]. Therefore, evidence guiding optimal treatment selection is limited.

SNM/SNS has emerged as an effective treatment for voiding dysfunction in recent years [3]. Studies have shown improvements in patient cohorts with DV, however, study populations are often not comparable due to inconsistencies in defining the subject population. Furthermore, different outcome measures of efficacy have been used such as symptom and quality of life scores, bladder diaries and measurements derived from urodynamics studies. An improvement in lower urinary tract (LUT) symptoms or dysfunction of more than 50% is often regarded as clinically significant, however, definitions vary across studies, as does the timing of assessment. Safety outcome measures such as complications, revision and explantation rates are also reported differently.

The aim of this review was to summarise the inconsistencies in definitions and studies evaluating SNM/SNS outcomes in adults with DV, and discuss the research required to address the gaps in our knowledge.

## 2 Methods

A multidisciplinary panel of experts participated in a discussion on DV at the International Consultation on Incontinence Research Society (ICI‐RS) held in Bristol, United Kingdom in June 2025. The panellists reviewed the definitions of DV in males and females, and discussed studies that evaluated SNM/SNS and factors that predict outcomes following this treatment. The panel reviewed urodynamic measurements/parameters and also patient co‐morbidities associated with greater complications, poorer outcomes and the need for re‐intervention. From the discussions at the meeting and subsequent e‐mail iterations, the panel proposed priority areas for further research.

### 2.1 Dysfunctional Voiding in Females

#### 2.1.1 Definition

The European Association of Urology (EAU) guideline for female non‐neurogenic LUT symptoms underlines the importance of making a distinction between voiding dysfunction and DV, which are two terms that are often incorrectly used interchangeably [1]. Voiding dysfunction is a general term, that can encompass several specific abnormalities affecting the process and efficiency of voiding. The International Continence Society (ICS) defines voiding dysfunction as “a diagnosis by symptoms and urodynamic investigations, defined as abnormally slow and/or incomplete micturition, based on abnormally slow urine flow rates and or abnormally high post‐void residuals”. Within the ICS definition it is pointed out that “pressure‐flow studies can be required to determine the cause of the voiding dysfunction” as there are clearly a number of abnormalities that can contribute to its occurrence [4]. DV is a more precise term which the ICS defines specifically as “a condition characterized by an intermittent and/or fluctuating flow rate due to involuntary intermittent contractions of the striated peri‐urethral or levator muscles during voiding in neurologically normal women” [5]. DV should therefore be considered as a distinct subtype of LUT dysfunction within the realm of voiding dysfunction.

Furthermore, a single unifying aetiology for DV is lacking, but rather a variety of abnormalities that may occur in isolation or combination [6]. Examples would include increased urethral sphincter activity or pelvic floor muscle contraction during voiding, which may themselves have a range of contributory factors. Current literature suggests that poorly learned toileting behaviour or a previous history of sexual abuse may be linked to this condition [7, 8].

It is evident that terminology is important within this topic area and subjects should be precisely defined to avoid heterogeneity within clinical research studies. Additional terms such as “poorly relaxing external sphincter”, “high tone non‐relaxing sphincter” or “Fowler's syndrome” are found in the literature, and though they are used in this paper, none have a standardised definition within ICS terminology documents.

#### 2.1.2 Phenotyping the LUT Dysfunction

Multimodality evaluation of suspected DV is almost always necessary. LUT symptoms assessment can suggest a possible diagnosis of DV, but in isolation is neither diagnostically accurate nor specific. Individuals most often describe voiding symptoms such as poor flow, hesitancy, intermittency and straining to void, however storage symptoms such as nocturia and urinary frequency and post micturition symptoms such as a sensation of incomplete bladder emptying are also reported, often secondary to poor bladder emptying. The evaluation of females with possible DV therefore necessitates uroflowmetry and urodynamic testing including filling and voiding measurements of pressure and flow, and additional tests such as external urethral sphincter (EUS) electromyography (EMG).

Urine flow studies characteristically show a fluctuating pattern which is often termed “staccato”, however further investigations are necessary before a diagnosis of DV can be made. Pressure‐flow studies may reveal a sustained or fluctuating detrusor pressure which rises as urine flow rate falls or stops. This pattern is described as distinctive for DV and similar to the urodynamic features seen in individuals with detrusor sphincter dyssynergia (DSD) [9]. The diagnosis of DV can however only be made in individuals without an underlying neurological condition.

Individuals with a primary disorder of urethral sphincter relaxation, sometimes called Fowler's syndrome, appear to have a slightly different urodynamic picture which includes reduced sensations during filling and a large capacity bladder. Urinary retention is likely to be arising from an exaggeration of the physiological guarding reflex, whereby excessive sphincter activity suppresses detrusor contractions [10]. Distinct urodynamic differences between DV and poorly relaxing external sphincter (PRES) have been reported, and the presence of detrusor overactivity during filling and greater detrusor contractility during voiding is suggested to be more in keeping with DV [11]. Videourodynamics may show a narrowing at the mid or distal urethra during voiding in females with DV, often with active sphincter contraction visualised in imaging.

Abnormalities in concentric needle EUS EMG were first identified in women with urinary retention in the 1980s [12]. Two abnormal EMG patterns were described: complex repetitive discharges (CRDs) ‐ repetitively firing, highly symmetric bursts of variable frequency with a distinctive “helicopter‐like” sound, and decelerating bursts, which have a myotonia‐like quality (pseudomyotonia) likened to the sound of whale noises. The relationship between urinary retention, abnormal EMG and EUS volume, measured with transvaginal or transrectal ultrasound, has been investigated in individuals diagnosed with Fowler's syndrome. Abnormal EMG was associated with an increased EUS volume, suggesting that an abnormality of the EUS plays a role in this condition [13, 14]. EMG shows increased external sphincter activity during voiding, often with bursts of activity synchronous with interrupted flow in case of DV.

Urethral pressure reflectometry is a technique allowing simultaneous measurements of cross‐sectional area and pressure in the urethra [15]. Cross‐sectional area is measured by acoustic reflectometry, while different preselected pressures are applied to the urethra. Although described over 20 years ago, it remains underutilized despite promising results and potentially greater reliability than urethral pressure profilometry. Whilst its role in evaluating DV in females is still unclear, high opening pressure might be expected in these individuals. This method is described as simple, and thus could play a role in the future diagnosis and management of EUS dysfunction, however, only after further research confirms its precise utility [15].

#### 2.1.3 Does LUT Dysfunction Phenotyping Help to Predict SNM/SNS Outcomes?

There is very little information in the literature regarding clinical, urodynamic and anatomical factors that can influence treatment outcomes in female DV. In a small study involving 15 women with either DV or unexplained voiding dysfunction, biofeedback was found to be more effective in women with inappropriate pubococcygeal muscle activation but not with poor sphincter relaxation [16]. This study highlights at least two distinct groups of women with DV, and concluded that the differentiation of the two groups was therapeutically relevant. The heterogeneity within this study population, comprising individuals with pelvic floor and sphincteric abnormalities, is a common finding in the literature. The precise phenotyping of individuals is recommended for future research as this would increase the level of certainty when investigating prognostic factors for treatment success.

Another study involving 62 individuals (30 with Fowler's syndrome based on characteristic EMG abnormalities and 32 with idiopathic retention) showed a high success rate in Fowler's syndrome treated with SNM/SNS, suggesting EUS EMG as a potential valuable tool for selecting candidates for this treatment [17]. However, these EMG abnormalities are not specific for the condition and CRDs have also been described in asymptomatic women, with variations linked to the menstrual cycle [18]. Despite this, the EMG abnormalities in individuals who are symptomatic could possibly predict treatment success following sacral neuromodulation.

### 2.2 Dysfunctional Voiding in Males

#### 2.2.1 Definition

DV is defined differently in males, and is characterized by an intermittent and/or fluctuating flow due to inadequate or variable relaxation generally of the sphincters during voiding in neurologically normal men where there is no historical, visible or measurable evidence of neurological disease [19]. The impact of other striated pelvic muscles such as levator ani or peri‐urethral muscles has not been considered in this definition [5, 19], and further work is needed to harmonise definitions so that there is a single definition. Excluding other causes for bladder outflow obstruction can be challenging since pressure‐flow studies alone may be unhelpful due to coexisting detrusor underactivity and other urological pathologies such as benign prostate enlargement.

Similarity between males and females is that the definition is limited to flow‐rate patterns. The flow obtained during pressure flow studies should be compared to free flowmetry to assess whether the finding of an abnormal flow is arising from unfamiliarity, or inhibition from having a catheter. Moreover, the type and grade of sphincter relaxation required for a normal voiding pattern has not been determined and needs to be researched [2].

#### 2.2.2 Phenotyping the LUT Dysfunction

DV is characterized by inappropriate or discoordinated contraction of the external urethral sphincter or pelvic floor during voiding, resulting in symptoms such as weak stream, hesitancy, straining and incomplete bladder emptying [2, 20]. Differentiating DV from other causes of LUT dysfunction such as benign prostate obstruction or detrusor underactivity is critical, and accurate phenotyping using videourodynamic studies, urethral pressure profilometry, symptom scores, bladder diaries and imaging can improve response to treatment and long‐term patient outcomes by better patient selection, thereby minimising unnecessary invasive interventions.

Urodynamic investigations, particularly pressure‐flow studies, can be combined with perineal surface EMG which serves as a proxy for urethral striated muscle activity, to help in identifying functional obstruction patterns and abnormal sphincter behaviour [21]. Videourodynamics, together with urethral pressure profilometry, can provide additional anatomical and functional insight, particularly in distinguishing DV from structural causes like bladder neck obstruction, and can help identify the level of the obstruction. Recent work from the Lower Urinary Tract Dysfunction Research Network (LURN) has emphasized the clinical importance of identifying symptom‐based phenotypes and latent class clusters in men with LUT symptoms, which may facilitate individualized management strategies [22].

Urethral sphincter dysfunction as well as bladder neck disease (BND) play an important role in male voiding dysfunction and should be suspected, especially in young and middle‐aged men with small prostate volumes (< 40 mL) not responding to α_1_‐adrenoceptor blocker treatment [23]. The combination of EMG and videourodynamics may identify more cases of DV than either modality alone, although there is no method to distinguish DV from neurogenic DSD in cases where occult neurogenic LUT dysfunction is suspected. Videourodynamics may show a wide‐open bladder neck which would help to differentiate DV from BND. However, there is very little data on what degree of bladder neck opening is considered predictive of developing voiding dysfunction [2, 24].

The measurement of EUS function in males is still a challenge. Combined pelvic floor EMG and videourodynamics assessment is considered superior to EUS pressure recordings for making a diagnosis [25]. However, a standardized cut‐off maximum urethral pressure closure (MUCP) value beyond which the pressure is deemed abnormally elevated is lacking. Moreover, videourodynamics with or without EMG is not widely available, nor consistently reliable, and therefore other diagnostic criteria are required. As an alternative, a predictive nomogram including a plateau pattern of detrusor contraction in association with the bladder outlet obstruction (BOO) index in the unobstructed range and smaller difference between Pdetmax and PdetQmax has been proposed. Nonetheless, a refinement in the definition of the plateau pattern of detrusor contraction and a validation of the proposed nomogram is still needed [26]. Based on the results of the analysis of videourodynamics parameters between individuals with BOO, it has been suggested that a cystometric bladder capacity “greater than 408.5 mL” could be a biomarker to differentiate subgroups with Poor Relaxation of the External Sphincter (PRES) from other types of voiding and storage male LUT dysfunction. However, a single parameter cannot be considered universally suitable for diagnosing DV. Furthermore, constructing a BOO risk score using variables such as age, symptom scores, uroflowmetry and prostate parameters to accurately predict DV has not been established, though it could be helpful to exclude benign prostate obstruction [27].

#### 2.2.3 Does LUT Dysfunction Phenotyping Help to Predict Treatment Outcomes?

Phenotyping LUT dysfunction in males with DV is essential to tailor effective treatments and improving patient outcomes. Phenotyping can also have a prognostic value, and individuals with isolated functional obstruction and preserved detrusor contractility may show better responses to conservative or targeted therapies such as pelvic floor biofeedback, pelvic floor relaxation or behavioural retraining, compared to those with anatomical obstruction or underactive bladder [28]. In addition, baseline detrusor pressure and the presence of co‐morbidities such as hypertension have been associated with differential treatment outcomes in females [29], and this may also apply to men, however needs further research. Studies evaluating outcomes following SNM/SNS are lacking in males.

### 2.3 Could Abnormal Central Regulation of LUT Networks Result in Dysfunctional Voiding?

Efforts to phenotype DV have conventionally been centred around recognising the pattern of LUT dysfunction. However DV may, in part, represent a problem of central coordination rather than purely peripheral or pelvic floor dysfunction. Neural control of urinary storage and voiding depends on balanced activity within forebrain storage and voiding networks, which converge largely onto the periaqueductal gray (PAG). The PAG is an important input nucleus to the pontine micturition centre (PMC) and the sum of the input to the PMC determines the switch between the storage and voiding phase of the LUT. Input from forebrain regions is critical for the voluntary switching between storage and voiding, but the mechanisms that delay voiding to maintain continence remain less well defined [30, 31]. If storage‐ and voiding‐related circuits are simultaneously or competitively active, the result may be impaired sphincter relaxation and incomplete emptying, as seen in DV. Functional neuroimaging studies suggest altered pontine control of pelvic floor muscles in individuals with DV compared to healthy individuals, in particular in the area of the PMC [32]. This reinforces the idea that central dysregulation contributes to this disorder. It remains to be investigated whether this change in PMC activity is due to alterations in processing of sensory information from the LUT, or alterations in top‐down control over the PMC.

SNM/SNS is thought to exert its effects at least partly through central mechanisms. By modulating afferent input, SNM/SNS may influence brainstem and forebrain circuits via the PAG and PMC, restoring appropriate network balance between storage and voiding [33]. Although speculative, SNM/SNS could restore abnormal co‐activation of storage and voiding pathways in DV, thereby normalizing pelvic floor and sphincter control and improving symptoms. Neuroimaging research can help to identify patient characteristics indicative of abnormal sensory processing to optimize patient selection for SNM/SNS.

### 2.4 Co‐Morbidities and SNM/SNS Outcomes

Patient characteristics and co‐morbidities may affect the efficacy of SNM/SNS, predispose to complications and affect whether individuals do well with this treatment. A holistic assessment of SNM/SNS outcomes therefore should integrate LUT dysfunction‐specific efficacy measures and complications, and this section reviews different patient‐related factors that can influence overall outcomes following SNM/SNS.

#### 2.4.1 Age

Age appears to influence outcomes following SNM/SNS. In females with nonobstructive urinary retention (NOUR) where an anatomical cause for obstruction has been excluded, increasing age is associated with reduced treatment success, with each 10‐year increment lowering the odds of response by 1.35 [34]. Similarly, Nasri et al. [35] reported a more favourable response in women with NOUR younger than 52 years. In males, the effect of age is more pronounced, and each 10‐year increase reduces the odds of success by 2.33 [34]. A favourable outcome has been observed in males with chronic urinary retention younger than a median age of 43 years [36]. Data in males and females with DV is lacking, however.

#### 2.4.2 Obesity

White et al. performed a prospective, longitudinal analysis of individuals with refractory LUT symptoms treated with staged SNM/SNS to identify predictors for adverse events [37]. Baseline body mass index (BMI) did not affect outcomes, however a change in BMI class whilst having the SNM/SNS implant from obese to normal, or morbidly obese to obese, was independently associated with developing pain at the site of implant, lead migration and trauma, and it was concluded that significant weight loss was contributory. A similar observation has been made by Peters et al. [38], and although the results initially suggested that reduction in BMI predicts reoperation, baseline BMI was not significant when adjusted for other covariates. In a cohort reported by Shih et al., no association could be established between reoperation and obesity [39]. Studies specifically evaluating BMI change in cohorts specifically with DV are lacking, however.

#### 2.4.3 Psychological and Neuropsychiatric Co‐Morbidities

The influence of psychological co‐morbidities on SNM/SNS outcomes remains unclear as mixed findings have been reported across studies. De Ridder et al. [17] and Peeters et al. [40] observed no correlation between psychiatric co‐morbidities and treatment response in females with Fowler's syndrome and mixed overactive bladder/non‐obstructive urinary retention, respectively. On the other hand, Marcelissen et al. [41] observed that a history of psychiatric disease predicted adverse events in a mixed overactive bladder/non‐obstructive urinary retention cohort. To the contrary, Coolen et al. [34] reported that in a large NOUR cohort undergoing stage 1 testing, individuals with a history of psychiatric illness, including posttraumatic stress disorder, showed better outcomes following SNM/SNS.

Individuals with functional neurological disorders experience neurological symptoms such as seizures, weakness, sensory loss, memory loss, speech disturbances and/or movement disorders which are incompatible with recognized neurological or medical conditions. These arise from an alteration in the processing of signals by brain networks rather than a change in the structure of the brain itself, however the exact cause for FND remains unknown [42]. FNDs have been reported in more than 50% of women with chronic idiopathic urinary retention, particularly with Fowler's Syndrome [43]. Outcomes of SNM/SNS in females with DV having FNDs is unknown, however three individuals were reported to have successful outcomes in a cohort with urinary retention. Patient selection criteria was not clearly defined in the study, though [44].

The occurrence of neuropsychiatric co‐morbidities can influence both the perception of LUT symptoms and outcomes of interventions. These co‐morbidities can affect coping strategies to chronic medical conditions such as DV, patterns of health‐seeking behaviour and engagement with urology teams [45]. Furthermore, it is possible that the specific neuropsychiatric conditions may themselves contribute directly to the LUT dysfunction in DV. For example, anxiety can enhance adrenergic activity and thereby increase the tone of the internal urethral sphincter or bladder neck. A general increase in striated muscle tone could involve the pelvic floor muscles and result in voiding difficulties similar to the pattern seen in DV.

#### 2.4.4 Opiate Use

Prescription opiate use has important implications on LUT functions and outcomes following SNM/SNS. It is speculated that opiate medications reduce detrusor activity and impair bladder sensation through μ‐ and δ‐receptor–mediated parasympathetic inhibition, while simultaneously increasing urethral sphincter tone via sympathetic overstimulation [46]. Clinically, high rates of prescription opiate use have been reported in individuals with Fowler's syndrome [47] and chronic idiopathic urinary retention [44]. Furthermore, chronic use of analgesic and antidepressant medication has been associated with a trend towards higher complication and reoperation rates following SNM/SNS [48].

## 3 Discussion

High‐quality studies evaluating the efficacy of SNM/SNS aim to generate clean datasets, and therefore individuals with significant co‐morbidities are frequently excluded. This limits the applicability of findings of these studies to real‐world practice and contributes to the uncertainty over which men and women with DV derive benefit from SNM/SNS. Published studies are heterogenous in different respects‐ poor patient phenotyping, lack of standardised assessment of LUT dysfunction, and insufficient evaluation of co‐morbidities‐ and therefore it is not possible to reliably predict treatment response with our current knowledge. These limitations underscore the need for a systematic assessment of predictive factors and are important areas for future research listed in Table 1.

**Table 1:** Research proposals to establish which individuals with Dysfunctional Voiding benefit from Sacral neuromodulation/sacral nerve stimulation.

Develop a tool through a cohort study that includes patient characteristics and co‐morbidities and urodynamics and EUS EMG findings which can be used to predict response of males and females with DV undergoing SNM/SNS. Investigate through an observational study whether noninvasive techniques can be developed to accurately determine which muscles are specifically affected in individuals with DV having non‐relaxing pelvic floor muscles, and evaluate whether a highly targeted physiotherapy protocol is more effective in improving symptoms. Investigate through a cohort study whether there is a relationship between resting pelvic floor muscle activity and flow patterns in individuals with DV. Explore through an experimental observational study whether there is a role for urethral pressure reflectometry in understanding the pathophysiology of DV and predicting outcomes following SNM/SNS. Establish through an RCT which therapeutic approaches could be offered to individuals with DV based on clinical features, urodynamic patterns and EUS EMG findings. Explore whether pelvic pain contributes to impaired voiding in individuals with DV having chronic pelvic pain, and to evaluate through an RCT whether intervening through pain management improve voiding functions. Assess through an observational study whether males with a previous history of surgery for bladder outflow obstruction respond differently to SNM/SNS. Explore whether there is a role for conditional stimulation, such as through high‐frequency pudendal nerve stimulation, for managing DV. Design an experimental study to evaluate whether alterations in forebrain and brainstem networks contribute to DV, particularly in terms of sensory processing and voluntary control. Design an experimental study to evaluate whether changes in pontine micturition centre activity in DV are primarily due to altered processing of LUT sensory input, or due to impaired top‐down control from forebrain regions. Explore if SNM/SNS influences central storage and voiding circuits, and whether this intervention can specifically restore the balance between storage‐ and voiding‐related pathways in individuals with DV. Investigate through a cohort study why outcomes of SNM/SNS are poorer with increasing age. Assess through an RCT whether SNM/SNS should be offered to individuals with DV who are likely to lose weight soon (e.g., if they are about to commence treatment with GLP1 analogues). Establish through an observational study whether individuals with DV taking prescription opiates benefit from SNM/SNS. Understand the impact of psychological comorbidities such as affective disorders and PTSD on SNM/SNS outcomes in individuals with DV. Explore through an observational study whether voiding difficulties improve in individuals with DV having functional neurological disorders undergoing SNM/SNS. Understand through an observational study whether anxiety in individuals with DV could result in an increased in generalised striated muscle tone, including the pelvic floor muscles, and thereby contribute to DV. Evaluate through a qualitative study design whether poor coping strategies and catastrophizing affect an individual′s perception of success following SNM/SNS. Investigate through a cohort study whether individuals with Ehlers Danlos Syndrome having DV respond differently to SNM/SNS. Investigate through an RCT whether SNM/SNS has a role in individuals with DV due to poorly relaxing pelvic floor muscles who have failed physiotherapy and other conservative measures?

Abbreviations: DV, dysfunctional voiding; EUS EMG, external urethral sphincter electromyography; LUT, lower urinary tract; RCT, randomised control trial; SNM, sacral neuromodulation; SNS, sacral nerve stimulation.

Precise phenotyping of DV, integrating clinical, urodynamic, neurophysiological and neuropsychiatric factors is essential for predicting which adults with DV respond favourably with SNM. Future research should focus on establishing criteria for patient selection and designing comprehensive prospective interventional studies to assess efficacy and complications.

## Conflicts of Interest

Jalesh N Panicker: Consultant (Idorsia, Coloplast, Medice), Speaker Honorarium (Coloplast, Wellspect) Chris Harding: consultancy and speaker fees from Viatris Pharmaceuticals, Astellas, Medtronic, Boston Scientific and Convatec. CH has received research grants from NIHR and The Urology Foundation. None of these are regarding the submitted work. Hashim Hashim: Consultant (Medtronic, Astellas), Speaker Honorarium (Astellas, Medtronic). Claire Hentzen: Consultant (Convatec, Coloplast, BBraun), Speaker Honorarium (Convatec, Hollister Inc, IPSEN, AbbVie). Nikita Bhatt: none declared. Arjun Nambiar: Consultant (Medtronic, AbbVie), Speaker honorarium (Merck). Brigitte Schurch: none declared. Mathijs de Rijk: No relevant conflicts of interest to disclose. Stefania Musco: Advisory board (Convatec Limited), Editorial Board (Pierre‐Fabre), Speaker Honorarium (Teleflex, Convatec Italia, B‐braun, Ecupharma, Hollister).

## Data Availability

Data sharing is not applicable to this article as no datasets were generated or analysed during the current study.
